# Secondary fracture prevention in primary care: a narrative review

**DOI:** 10.1007/s00198-024-07036-1

**Published:** 2024-04-23

**Authors:** Mawson Wang, Markus J. Seibel

**Affiliations:** 1 Bone Research Program, ANZAC Research Institute, The University of Sydney at Concord Campus, Hospital Rd, Gate 3, Concord, NSW Australia; 2 Department of Endocrinology and Metabolism, Concord Hospital, Sydney Local Health District, Concord, NSW Australia

**Keywords:** Fracture liaison service, Fracture prevention, Osteoporosis, Primary care, Secondary fracture prevention

## Abstract

The global burden of osteoporosis continues to rise with an ageing population. Untreated osteoporotic fractures not only heighten the risk of subsequent fractures but are associated with excess mortality. Although primary care guidelines consistently stress the importance of secondary fracture prevention, fewer than 20% of patients are appropriately treated for osteoporosis following an initial osteoporotic fracture. This worldwide phenomenon is known as the osteoporosis care gap. This literature review examines the barriers to secondary fracture prevention in primary care and evaluates the effectiveness of targeted primary care interventions. Common themes emerged from the majority of qualitative studies, including a need for improved communication between the hospital team and primary care, better defined responsibilities and osteoporosis-directed education for the primary care physicians. Quantitative studies demonstrated that most targeted, intensive interventions aimed at educating patients and their primary care physician about osteoporosis treatment significantly increased rates of investigation and treatment. Greater uptake of models of secondary fracture prevention in primary care is urgently needed to address the osteoporosis care gap.

## Introduction

Osteoporosis is a systemic disorder characterized by loss of bone mass and microarchitectural deterioration leading to low bone mineral density (BMD) and increased risk of a minimal trauma, or fragility fracture. It is the most common bone disease, affecting over 200 million people globally [1]. Its prevalence is highest in the elderly, where one in three women and one in five men over the age of 50 will experience an osteoporotic fracture in their remaining lifetime [2]. As the elderly population is expected to double by 2050, this will lead to an unprecedented increase in the global burden of osteoporosis and resultant fragility fractures [3]. The impending wave of osteoporosis and its impact on global healthcare cannot be underestimated. A concerted and proactive effort among policymakers, medical professionals and the public is imperative to confront this escalating challenge.

### Global prevalence and socioeconomic burden

The global prevalence of osteoporosis has been estimated from a recent systematic review and meta-analysis across 86 studies to be 23.1% in women and 11.7% in men [4] although notably the diagnostic criteria for osteoporosis was not consistent across all studies. The Global Burden of Disease Fracture Study estimated 178 million new fractures in 2019 alone, an increase of over 33% since 1990 [5]. The regions with the highest age-standardized incidence rate of fractures were in Australasia, central and eastern Europe, and the lowest incidence rates in sub-Saharan Africa [5]. Within Australia, the estimated prevalence of self-reported osteoporosis was 15% in women and 3% in men according to the Australian Health Survey in 2011–2012, while the prevalence of BMD-defined osteoporosis was 23% in women and 6% in men over the age of 50 in the Geelong Osteoporosis Study [6, 7]. The Burden of Disease Report on osteoporosis estimates that in 2022, 6.2 million Australians over the age of 50 suffer from osteoporosis or poor bone health, representing a 31% increase over the preceding decade [8]. At a socioeconomic level, the burden of osteoporotic fractures is enormous. Up to 40% of patients are unable to mobilize independently, and 33% are completely dependent or require nursing home residence at 1 year post hip fracture [9, 10]. The economic cost associated with osteoporosis is estimated at around 3.4 billion AUD per year in Australia in 2017 [11], 57 billion EUR per year in Europe in 2019 [12] and 25 billion USD per year in the United States by 2025 [13]. These numbers will continue to rise as the incidence of osteoporosis increases.

### Prevalence of osteoporosis in primary care

The prevalence of BMD-defined osteoporosis in a large Netherlands primary care practice of 712 postmenopausal females was 7% [14]. Similarly, reviews of primary care databases yielded a prevalence rate of 6.8% in a Belgian study of 543 patients aged over 65 years [15], and up to 11% in a US study of 389 women aged over 50 years [16]. In the largest US study utilizing a primary care clinical database of over 660,000 patients aged over 18 years, the prevalence of osteoporosis and osteopenia was 6.6% [17]. A similar Australian study analyzing a national primary care database of over 200,000 patients aged 50 years or older estimated the prevalence of osteoporosis to be 12.4% [18].

### Importance of secondary fracture prevention and the osteoporosis care gap

Fragility fractures, if left untreated, significantly increase the risk of subsequent fractures. This risk is highest in the first few years following an initial fracture and gradually declines over time [19–23]. Additionally, a recent cohort study has shown that subsequent fracture risk is increased after any clinical fracture, not just osteoporotic fractures [24]. Bone mineral density declines at a greater rate following a hip fracture compared to those without a fracture [25, 26], as does physical performance [27] and quality of life [28]. Strategies for the early identification and management of incident fractures should therefore be a priority for all clinicians, as clinical inertia may lead to subsequent fractures over the following years. Mortality is increased following any fracture, particularly hip fractures which have 1-year mortality rates exceeding 20% [29–32], but also for non-hip fractures, to a lesser extent [33, 34]. Importantly, subsequent fracture was associated with a twofold increase in 5-year mortality for women and threefold for men [34]. The excess mortality following a refracture was higher than the mortality attributed to the first fracture and persisted for up to 10 years.

The risk of falls and fear of falling is increased following an initial fracture, and incident falls are a strong predictor of subsequent fracture risk [35–38]. Interventions including targeted exercises, patient education, assistive technology, environment modifications and falls assessments were associated with a reduction in falls and resultant fractures [39]. Primary care plays a crucial role in screening for falls risk: evaluating gait, strength and balance; addressing risk factors and medical comorbidities (including laboratory and BMD testing); and referring to allied health and other specialists [40]. Falls assessment and management thus play a crucial role in secondary fracture prevention and should be considered by all clinicians involved in osteoporosis-related care, including primary care.

Current guidelines for primary care physicians (PCPs) convey a consistent message regarding secondary fracture prevention: that incident fragility fractures warrant immediate investigation and treatment. Osteoporosis guidelines from the UK, Europe and the USA all support that individuals with a history of fragility fracture, particularly hip or vertebral fractures, are presumed to have osteoporosis, considered high risk of sustaining future fractures and recommended pharmacotherapy without needing to confirm low BMD [41–43]. The Royal Australian College of General Practitioners (RACGP) guidelines additionally recommend BMD testing in those with non-hip, non-vertebral fractures and to initiate treatment if T-scores ≤  − 1.5 SD [44].

However, despite these clear guidelines and the availability of safe and effective pharmacotherapies that reduce the risk of follow-up fractures, fewer than 20% of patients who have suffered a first osteoporotic fracture are appropriately treated for osteoporosis. This gap in osteoporosis management is a global phenomenon and has been documented in numerous studies from Canada, the USA, Denmark, Spain, South Korea and Australia [45–50]. Eisman et al. conducted a survey involving over 69,000 patients in Australian primary care and found that among women > 60 years attending their PCP, 29% had reported previous fractures but less than one-third were on osteoporosis treatment, but only 40% were informed they had osteoporosis [51]. In another Australian primary care survey with over 37,000 patients, 12.6% were reported as having a fracture (17.4% in women) of which only 29.9% were on osteoporosis treatment [50]. A Canadian population study found that while rates of post-fracture treatment and BMD testing initially increased between 1996/1997 and 2003/2004, it had declined again by 2007/2008 where less than 15% of patients received adequate intervention [52]. This systematic failure in adequately addressing and treating patients who have sustained an osteoporotic fracture is known as the ‘osteoporosis care gap’.

### Barriers to secondary fracture prevention in primary care

Despite the known deleterious sequelae of minimal trauma fractures, osteoporosis is severely under-recognized and undertreated in primary care. The reasons for this are multifactorial and can be summarized into several main themes.

Firstly, there is ambiguity concerning who is responsible for osteoporosis management due to poor communication between the hospital and primary care provider. Following a fracture, hospital-based specialists may assume that the patient’s PCP will review and initiate osteoporosis treatment post-discharge, whereas the PCP may only do so if specifically directed by the hospital specialists, thus leading to a cycle where neither clinician initiates treatment [53]. PCPs also do not reliably receive discharge summaries or correspondence from the hospital and may not even be aware of the event [54]. Bennett et al. interviewed PCPs and hospital staff involved in secondary fracture prevention programs (SFPP) and learned that PCPs often found it difficult to contact or direct enquiries to SFPP staff. Conversely, staff often questioned whether their correspondence reached the intended PCP [55].

Clinician-related factors pertaining to lack of knowledge or confidence with osteoporosis management are key barriers to secondary fracture prevention. Numerous worldwide studies have demonstrated knowledge deficiencies in PCPs related to osteoporosis, and in particular, pharmacotherapy [56–58]. In an Australian primary care study, it was found that the decision to treat or not treat patients was unrelated to the presence of major osteoporosis risk factors which implies that the management of osteoporosis is non-systematic and inconsistent with local primary care guidelines [59]. Conversely, Singaporean PCPs who self-reported good guideline knowledge were more likely to report confidence with initiating osteoporosis treatment [60].

A prevailing theme that persists across healthcare providers and patients is the perception of osteoporosis being a ‘silent’ disease and of ‘low priority’ in comparison to other medical comorbidities [18, 56, 61]. There is little urgency in managing and treating osteoporosis with ‘preventative’ medicines, and some PCPs may opt to monitor rather than initiate treatment, even after patients have sustained a fracture [18]. Patients who do not perceive the medications to be effective in reducing fracture risk are less likely to commence bisphosphonate treatment after a fracture [62].

Medication-related factors may prevent the initiation of pharmacotherapy, due to medical contraindications, comorbidities or rare side-effects such as osteonecrosis of the jaw. For example, bisphosphonate prescriptions fell significantly in the 9 months following extensive media coverage on osteonecrosis of the jaw [63]. The cost of medications and limited availability of medications at the practice were also cited as common barriers to treatment in a Singaporean PCP survey [60].

Finally, a lack of financial incentive for investigating and treating osteoporosis may pose an additional barrier in certain healthcare settings. In a Swedish PCP survey, it was reported that investigations which did not receive financial reimbursement from the healthcare system (as is the case for osteoporosis) were not encouraged to be performed [61].

### The interface between hospital-based services and primary care

Over the past two decades, there has been significant progress in implementing systematic and coordinated models of care for secondary fracture prevention in hospital settings worldwide [64–66]. These SFPPs, commonly known as Fracture Liaison Services (FLS), involve a structured approach to the diagnosis, management and follow-up of patients with an osteoporotic fracture, typically overseen by a dedicated coordinator [67, 68]. Ganda et al. analyzed different models of care for secondary fracture prevention and divided these models into four categories of intensity: Type A, identification, assessment and treatment of patients happens within the service; Type B, identification and assessment happens within the service but treatment initiation is left to the primary care physician; Type C, patient and PCP education only; Type D, patient education only [69]. The more intensive models of care led to statistically significantly higher rates of both BMD testing and treatment initiation. While type A models have been shown to be effective in preventing secondary fractures, with some studies suggesting that these services are also cost-effective [69–73], hospital-based SFPPs have several limitations. Firstly, they only capture patients who seek hospital-level care. Thus, these services often miss patients who do not get admitted, present to their PCP with minor fractures, or have asymptomatic vertebral fractures. Furthermore, most hospital services are limited by their low capacity and are unable to meet the demands of the increasing number of patients with osteoporosis. Finally, there is inconsistent and insufficient communication and integration with primary care, which introduces inefficiencies and errors.

Primary care plays a central role in addressing and managing secondary prevention of numerous chronic diseases, including osteoporosis. There is an increasing trend of outpatient visits to PCPs for osteoporosis-related care which partly reflects the increasing burden of osteoporosis in the community [74]. Primary care has the potential to manage much higher numbers of patients with osteoporosis than resource-limited hospital-based FLS, but for this to occur successfully there needs to be: (1) effective methods of detecting patients in need of secondary fracture prevention, (2) clear communication between the FLS and primary care, and (3) appropriate management of patients according to established guidelines.

Numerous qualitative studies have explored the issues, barriers, and supports in the management of secondary fracture prevention in primary care. Quantitative studies have examined the efficacy of interventions aimed at improving the detection and treatment of osteoporosis by PCPs post-fracture, often coordinated by an FLS. The following literature review aims to summarize and evaluate these studies.

## Results

### Qualitative studies

A Spanish group of authors reviewed current hospital-based FLS practices, specifically their integration with primary care [75]. While three-quarters of FLS had pathways for communication with primary care through email, telephone, fax or virtual care, only 25% assigned a designated coordinator to manage this communication with primary care. In most cases, a clinical report was provided to the PCP (sometimes via the patient). One-third of FLS shared common software with the PCP, which increased the likelihood of the PCP receiving relevant information. The authors proposed several strategies, including expanding communication methods with PCP, standardization of the FLS report, phone calls to monitor patient adherence, training sessions for the PCP and performance indicator monitoring at the centres. An Australian qualitative study of PCPs found that timeliness and accessibility of clinical correspondence from the FLS were identified as one of the top priorities, and electronic delivery was the preferred mode of information transfer [55]. The importance of communication is again stressed by Meadows et al. who concluded that until all stakeholders acknowledge and act upon the ‘integral role of communication’, this will remain an issue [54].

In a UK study, over half of surveyed PCPs expressed a preference for initiation of treatment by the FLS, as it would reassure them that the patients have had ‘full counselling’ from specialists [76]. This was echoed in a Swedish study, where time-poor PCPs preferred that district nurses played a larger role in performing fracture risk assessment and management [61]. A Singaporean SFPP (OPTIMAL) found that discharging patients back to primary care remained inadequate in their patient cohort and urged the development of better transition programs [77]. A planned Australian mixed-methods study aims to develop a new post-fracture model of care in the primary care setting to improve osteoporosis diagnosis and treatment [53]. The study will involve interviews with PCPs and patients to identify their attitudes and needs towards osteoporosis care and utilize co-design workshops with consumers and stakeholders to create this new model. Possible components of this intervention may include adapting the FLS to suit the primary care setting, PCP educational programs, and greater use of electronic reminders and clinical decision tools. Conclusions drawn from numerous studies of PCP interviews have reiterated the importance of increasing PCP and patient knowledge about the importance of secondary fracture prevention and addressing expected barriers [55, 62]. These concepts have been incorporated into the design of numerous quantitative studies.

### Quantitative studies

Numerous studies have examined the effectiveness of interventions which target PCPs and/or patients to bring their attention to the patient’s recent fracture and the need for further assessment and treatment (Table 1). A systematic review and meta-analysis of 13 studies examining interventions which improve osteoporosis management in primary care found that most approaches were multifaceted and involved a combination of sending PCP notifications, providing patients with educational material, providing PCPs with osteoporosis training and phoning patients to ensure initiation of management [78]. Compared to no intervention, targeted interventions led to an absolute difference of 22–51% in the incidence of BMD testing and 18–29% in the incidence of treatment initiation. Despite differences in healthcare systems, the following studies all share common themes in their concerted approach to secondary fracture prevention in the acute post-fracture setting.

**Table 1:** Summary of quantitative studies

Study	Patient inclusion/exclusion criteria	Intervention groups	Outcomes and method of outcome assessment
Roux et al. 2013 (Canada)	Patients ≥ 50 years with fragility fracture (FF) Non-osteoporotic fractures, hip fractures (received evaluation and treatment), CKD Stage 4–5, hyperparathyroidism, multiple myeloma, metastatic bone disease	Minimal intervention group (*n* = 370): ▪ Patient education (verbal and written): osteoporosis information and importance of PCP follow-up ▪ Letter to PCP: patient’s FF, rationale for osteoporosis treatment ▪ Follow-up patient phone calls: 6 and 12 months ▪ Safety net: if patients untreated at 6 months, reminder letter sent to PCP, if untreated at 12 months, intervention letter sent to PCP to advise treatment Intensive intervention group (*n* = 311): ▪ Patient education (verbal and written): osteoporosis information and importance of PCP follow-up ▪ Letter to PCP: patient’s FF, rationale for osteoporosis treatment ▪ Investigations: screening blood test and BMD test prescribed with results sent to PCP. Abnormal results led to individualized letter to PCP ▪ Follow-up patient phone calls: 4, 8 and 12 months ▪ Safety net: if patients untreated at 4 and/or 8 months, intervention letter sent to PCP to advise treatment Control group (*n* = 200): ▪ Follow-up patient phone calls: 6 and 12 months (without mention of osteoporosis) Safety net: if patients untreated at 12 months, verbal education provided and intensive intervention offered	Rate of osteoporosis treatment at 12 months: ▪ Change in treatment rate from baseline rate: + 31.6% in minimum group (*p* < 0.001 vs. control), + 42.6% in intensive group (*p* < 0.0001 vs. control), + 16.1% in control group ▪ Among those initially untreated at baseline: 40.4% in minimum group (*p* < 0.0001 vs. control), 53.2% in intensive group (*p* < 0.0001 vs. control), 18.8% in control group Confirmation with patient’s pharmacist
Jaglal et al. 2012 (Canada)	Patients ≥ 40 years with fracture of hip, forearm, wrist, rib, sternum, thoracic and lumbar spine, shoulder and upper arm, pelvis, lower leg and ankle Fracture not due to fall from standing height, nursing home residents, or fractures occurring > 3 months ago	Intervention group (*n* = 130): ▪ Patient education (verbal and written): osteoporosis information, importance of PCP follow-up and need for BMD test ▪ Letter to PCP: patient’s FF, rationale for osteoporosis treatment, pocket cards for osteoporosis screening and treatment ▪ Follow-up patient phone calls: 3 months Control group (*n* = 137): Patient education (verbal and written): falls prevention	Rate of osteoporosis treatment at 6 months OR if normal BMD, prevention advice given: ▪ 45% in intervention group vs. 26% in control group (*p* = 0.003) Rate of BMD test scheduled or performed at 6 months: ▪ 57% in intervention group vs. 21% in control group (*p* = < 0.0001) Interview with patient
Cranney et al. 2008 (Canada)	Postmenopausal women with wrist fracture Traumatic fracture, current osteoporosis treatment except hormone replacement therapy	Intervention group (*n* = 125): ▪ Patient education (written): letter sent 2 weeks and 2 months after fracture, checklist of risk factors to calculate risk score, osteoporosis information and importance of PCP follow-up ▪ Letter to PCP: letter sent 2 weeks and 2 months after fracture, patient’s FF, rationale for osteoporosis treatment, treatment algorithm Control group (*n* = 145): No additional communication^1^	Rate of osteoporosis treatment at 6 months: ▪ 28% in intervention group vs. 10% in control group (*p* = 0.002) Rate of BMD test performed at 6 months: ▪ 53.5% in intervention group vs. 25.5% in control group (*p* < 0.0001) Interview with patient
Bessette et al. 2011 (Canada)	Women ≥ 50 years with FF of wrist, forearm, humerus, scapula, clavicle, sternum, thoracic or lumbar vertebrae, pelvis, sacrum, hip, femur, proximal and distal tibia, fibula and foot Cervical spine, skull or face, hand or finger, toe, foot or patella, pathological fractures, nursing home residents, participation in another osteoporosis trial	Written material group (*n* = 379): ▪ Patient education (written): osteoporosis information, importance of PCP follow-up and need for BMD test ▪ Letter to PCP: rationale for osteoporosis treatment Video and written material group (*n* = 409): ▪ Patient education (15 min video): osteoporosis information, importance of PCP follow-up and need for BMD test, non-pharmacological therapies, falls prevention ▪ Patient education (written): osteoporosis information, importance of PCP follow-up and need for BMD test ▪ Letter to PCP: rationale for osteoporosis treatment Control group (*n* = 386): No additional communication	Rate of osteoporosis treatment at 12 months: ▪ 11% in video and written material group (*p* = 0.06 vs. control), 12% in written material group (*p* = 0.05 vs. control) and 8% in control group Rate of BMD test performed at 12 months ▪ 16% in video and written material group (*p* = 0.04 vs. control), 15% in written material group (*p* = 0.07 vs. control) and 12% in control group Interview with patient
Leslie et al. 2012 (Canada)	Patients ≥ 50 years with fracture of hip, spine, humerus or forearm Current osteoporosis treatment, previous fracture within 12 months of recent fracture, BMD test within 3 years of recent fracture, nursing home residents	Physician and patient notification intervention group (*n* = 1421): ▪ Patient education (written): osteoporosis information, importance of PCP follow-up ▪ Letter to PCP: patient’s FF, rationale for osteoporosis treatment, BMD request form Physician notification intervention group (*n* = 1363): ▪ Letter to PCP: patient’s FF, rationale for osteoporosis treatment, BMD request form Control group (*n* = 1480): No additional communication	Rate of osteoporosis treatment at 12 months: ▪ 16.5% in physician and patient group, 14.7% in physician group, 10.6% in control group (p values not available) Rate of BMD test performed at 12 months: ▪ 18.2% in physician and patient group, 16.4% in physician group, 3.9% in control group (*p* values not available) Data linkage with healthcare database
Majumdar et al. 2004 (Canada)	Patients ≥ 50 years with wrist fracture Current osteoporosis treatment, admitted patients, nursing home residents	Intervention group (*n* = 55): ▪ Patient education (written): osteoporosis information ▪ Patient education (verbal): osteoporosis information, importance of PCP follow-up ▪ Letter to PCP: patient’s FF, rationale for osteoporosis treatment Control group (*n* = 47): ▪ Letter to PCP: patient’s fracture (without mention of osteoporosis) and clinic follow-up plans Patient education (verbal and written): falls prevention	Rate of osteoporosis treatment at 6 months: ▪ 40% in intervention group vs. 10% in control group (*p* = 0.002) Rate of BMD test performed at 6 months: ▪ 62% in intervention group vs. 17% in control group (*p* < 0.001) Interview with patient and confirmation with patient’s pharmacist
Majumdar et al. 2007 (Canada)	Patients ≥ 50 years undergoing hip fracture surgery Current osteoporosis treatment, nursing home residents, pathological fractures	Intervention group (*n* = 110): ▪ Patient education (written): osteoporosis information, importance of PCP follow-up ▪ Patient education (verbal): importance of BMD testing ▪ Arrangement of BMD test and result review ▪ Prescription of oral bisphosphonate if low BMD Control group (*n* = 110): ▪ Patient education (written): osteoporosis information, importance of PCP follow-up ▪ Patient education (verbal): falls prevention, importance of calcium and Vitamin D intake	Rate of osteoporosis treatment at 6 months (post-intervention): ▪ 54% in intervention group (*p* < 0.001 vs. control), 38% in facilitated intervention group (*p* < 0.001 vs. control), 22% in control group Rate of BMD test performed at 6 months (post-intervention): ▪ 80% in intervention group (*p* < 0.001 vs. control), 68% in facilitated intervention group (*p* < 0.001 vs. control), 29% in control group Rate of appropriate care (BMD performed and treatment started in those with low BMD) at 6 months (post-intervention): ▪ 71% in intervention group (*p* < 0.001 vs. control), 45% in facilitated intervention group (*p* < 0.001 vs. control), 26% in control group Interview with patient
Morrish et al. 2009 (Canada)	Second phase of above study	In second phase of the study (6 months post initial intervention), the control group was enrolled in facilitated intervention group (*n* = 110): ▪ Arrangement of BMD testing Letter to PCP with BMD test result	
Hawker et al. 2003 (Canada)	Patients ≥ 40 years with fracture of wrist, hip, ankle, vertebrae or humerus Traumatic fracture, current osteoporosis treatment	Intervention group (*n* = 139): ▪ Patient education (verbal): osteoporosis information, importance of PCP follow-up ▪ Letter to PCP (given to patient, not PCP): patient’s FF, rationale for osteoporosis treatment Control group (*n* = 139): No additional communication	Rate of any treatment at 3 months for intervention, 3–9 months for controls: ▪ 24.4% in intervention group vs. 31.4% in control group (OR 1.04, CI 0.53–2.03) Rate of BMD test performed at 3 months for intervention, 3–9 months for controls: ▪ 81.7% in intervention group vs. 92% in control group (OR 0.26, CI 0.14–0.51) Interview with patient
Gardner et al. 2005 (USA)	Patients ≥ 65 years admitted with hip fracture Current osteoporosis treatment, traumatic fracture, history of alcoholism or dementia	Intervention group (*n* = 36): ▪ Patient education (verbal): 15 min face-to-face, osteoporosis information, importance of PCP follow-up, question list for PCP ▪ Follow-up patient phone calls: 6 weeks Control group (*n* = 36): Patient education (written): falls prevention with brief mention of osteoporosis	Rate of osteoporosis ‘being addressed’ at 6 months: ▪ 42% in intervention group vs. 19% in control group (p = 0.036) Interview with patient
Rozental et al. 2008 (USA)	Women ≥ 50 years and men ≥ 65 years with radius fracture Current osteoporosis treatment, BMD test within 2 years of recent fracture, traumatic fracture	Intervention group 1 (*n* = 27): ▪ Arrangement of BMD testing ▪ Letter to PCP: BMD test result Intervention group 2 (*n* = 23): ▪ Letter to PCP: patient’s FF, rationale for osteoporosis treatment No control group	Rate of osteoporosis treatment at 6 months: ▪ 74% in intervention group 1 vs. 26% in intervention group 2 (*p* < 0.001) Rate of BMD test performed at 6 months: ▪ 93% in intervention group 1 vs. 30% in intervention group 2 (*p* < 0.001) Interview with patient and review of medical records
Skedros, 2004 (USA)	Patients ≥ 50 years with FF Current osteoporosis treatment, nursing home residents, cognitive impairment, requiring support devices	Intervention group (*n* = 69) ▪ Patient education (verbal): osteoporosis information, importance of PCP follow-up ▪ Letter to PCP: patient’s FF, rationale for osteoporosis treatment No control group	Rate of PCP follow-up at 3 months: 56.5% Rate of osteoporosis treatment (out of those who followed up) at 3 months: 56.4% Rate of BMD test performed (out of those who followed up) at 3 months: 43.6% Method of outcome assessment not specified
Feldstein et al. 2006 (USA)	Women aged 50–89 years with FF Fractures of skull, face, fingers, toes, ankle or open fracture suggestive of trauma, current osteoporosis treatment, previous BMD test, nursing home residents, malignancy, chronic renal failure, dementia, organ transplant, cirrhosis, participation in another osteoporosis trial	EMR and patient notification intervention group (*n* = 113): ▪ Patient education (written): osteoporosis information, importance of PCP follow-up ▪ EMR notification to PCP: patient’s FF, rationale for osteoporosis treatment, treatment guidelines ▪ Follow-up EMR reminder: 3 months if no treatment or BMD test is ordered EMR notification intervention group (*n* = 107): ▪ EMR notification to PCP: patient’s FF, rationale for osteoporosis treatment, treatment guidelines ▪ Follow-up EMR reminder: 3 months if no treatment or BMD test is ordered Control group (*n* = 107): No additional communication	Rate of osteoporosis treatment or BMD test performed at 6 months: ▪ 43.1% in EMR and patient intervention group (*p* < 0.001 vs. control), 51.5% in EMR intervention group (*p* < 0.001 vs. control, 5.9% in control Data linkage with healthcare database
Wood et al. 2017 (UK)	Patients admitted with neck of femur fracture	Pre-intervention group (*n* = 22): ▪ Patient reviewed as suitable for denosumab while inpatient ▪ No standardized wording of denosumab recommendation for PCP on discharge summary Intervention group (*n* = 30): ▪ Patient reviewed as suitable for denosumab while inpatient ▪ Documentation of ‘denosumab- GP to prescribe and start on discharge’ on hospital notes intended to be included in discharge summary Letter to PCP: recommendation for denosumab	Rate of denosumab treatment (no clear timeframe): ▪ 22% in pre-intervention group vs. 74% in post-intervention group Confirmation with patient’s PCP
Naranjo et al. 2014 (Spain)	Patients ≥ 50 years with FF Fractures of face, skull, ribs, hands, feet, pathological fracture, severe functional disability, advanced liver disease, renal failure, serious medical illness	Intervention group (*n* = 330): ▪ Patient education (verbal): face-to-face, osteoporosis information, importance of PCP follow-up, bisphosphonate education ▪ Prescription of oral bisphosphonate based on criteria ▪ Arrangement of BMD testing ▪ Letter to PCP: BMD test result ▪ PCP education: rheumatologist-led 60 min session, explanation of study, rationale for osteoporosis treatment No control group	Rate of bisphosphonate continuation in those prescribed bisphosphonate at 3 months: 78% Interview with patient and review of medical records
Sanfelix-Genoves et al. 2010 (Spain)	Patients ≥ 50 years receiving healthcare as part of longitudinal observational prospective cohort study (ESOSVAL-R)	Intervention group (*n* = 400 general practices): ▪ EMR improvement: new follow-up sheet, risk factor variables for osteoporosis patients ▪ PCP education: 4 h classroom session, online education Control group (*n* = 400 general practices): EMR improvement: new follow-up sheet, risk factor variables for osteoporosis patients	Rate of ‘appropriate treatment’ according to established national and international osteoporosis guidelines Rate of patients ≥ 70 years diagnosed with osteoporosis and/or FF with osteoporosis treatment compared to all people ≥ 70 years in the practice ▪ Follow-up ongoing Data linkage with healthcare database
Kessous et al. 2014 (Israel)	Women aged 48–70 years with radius fracture Current treatment for or previous diagnosis of osteoporosis, tumour-related pathological fractures, chronic steroid use	Intervention group (*n* = 35): ▪ Patient education (written): osteoporosis information ▪ Patient education (verbal): questionnaire about osteoporosis knowledge (also in control group) ▪ Letter to PCP: rationale for osteoporosis treatment Control group (*n* = 35): Baseline questionnaire which asked about osteoporosis knowledge and prior workup and mentioned possible link between FF and osteoporosis	Rate of referral to PCP at 6–8 weeks: 68.6% in intervention group vs. 22.9% in control group (*p* = 0.001) Rate of BMD test performed at 6–8 weeks: 40% in intervention group vs. 14.3% in control group (*p* = 0.001) Interview with patient
Goldshtein et al. 2020 (Israel)	Patients with either recorded T-score ≤ − 2.5 SD (primary prevention subgroup) or femoral neck or clinical vertebral fracture (secondary prevention subgroup) Current osteoporosis treatment, traumatic fracture	Pre-intervention group (unclear number): ▪ No physician alert notification Intervention group (*n* = 21,070): ▪ EMR alert to PCP, endocrinologist or geriatrician: EMR pop-up alert with ‘smart-set’ screens to order blood tests, order prescriptions for osteoporosis treatment, refer to specialists or allied health, print information page for patient	Time from event until initiation of osteoporosis treatment: Time to treatment initiation shorter in post-intervention group than pre-intervention group for secondary prevention subgroup (*p* < 0.001) Rate of osteoporosis treatment at 6 months: Post-intervention 27.7% vs. pre-intervention 12.3% for hip fracture subgroup (*p* < 0.001) Post-intervention 27.1% vs. pre-intervention 17.4% for vertebral fracture subgroup (*p* < 0.001) Data linkage with healthcare database
Bliuc et al. 2006 (Australia)	Patients ≥ 20 years with FF Current osteoporosis treatment, traumatic fracture, finger or toe fractures	Letter and BMD test group (*n* = 79): ▪ Patient education (written): osteoporosis information, importance of PCP follow-up ▪ Offer of BMD test with results sent back to patient to bring to PCP Letter group (*n* = 75): ▪ Patient education (written): osteoporosis information, importance of PCP follow-up	Rate of osteoporosis treatment at 6 months: Letter and BMD test group 5% vs. letter group 7% (no diff.) Rate of BMD test performed at 6 months: Letter and BMD test group 38% vs. letter group 7% (*p* = 0.001) Interview with patient
Wang et al. (in progress) (Australia)	Patients ≥ 50 years referred for a scan by their PCP to a private radiology practice) or a hospital-based FLS with a FF diagnosed by natural language processing technology Traumatic or pathological fracture, complex or major fractures (FLS only), fractures of toes, fingers, face, ankle (women only), current osteoporosis treatment, pregnant women	Intervention group (*n* = 600): ▪ Alert to PCP: electronic and/or fax alert informing PCP of recent FF, rationale for osteoporosis treatment, osteoporosis guidelines, post-treatment surveys, reminder alerts Control group (*n* = 600): ▪ No additional communication	Rate of osteoporosis treatment at 3 months Rate of BMD test performed at 3 months Rate of chronic disease management plan initiation at 3 months Rate of osteoporosis-related blood test at 3 months Rate of continued prescription of osteoporosis treatment at 9 months Data linkage with healthcare database

^1^No additional communication refers to no additional osteoporosis-directed intervention. Patients received usual care and their PCP may receive a discharge report from the hospital in relevant studies. Patients were contacted to provide consent for the study and to obtain outcome data

#### North America

Most PCP-targeted interventions in secondary fracture prevention have arisen from Canada. Roux et al. targeted patients aged > 50 years in an orthopaedic clinic who had sustained a fragility fracture, applying and analyzing one of the most intensive interventions of any trials performed in this area [79]. Patients were randomized to a minimal intervention, intensive intervention or control group. The minimal intervention group (*n* = 370) received verbal and written education from a coordinator about the causal link between a fragility fracture and osteoporosis, and a standard letter notified their PCP of the fracture, treatment rationale and recommended investigations and treatment. Reminder letters were sent to their PCP if they remained untreated after 6 months, and an intensive intervention was proposed if untreated after 12 months. Patients in the intensive intervention group (*n* = 311) received the same interventions as the minimal intervention group, with additional screening blood tests, coordinators following up on abnormal results and a written prescription for a BMD scan. Telephone follow-ups occurred at 4, 8 and 12 months, and PCPs were advised in writing to treat for osteoporosis if patients remained untreated after 4 or 8 months. The control group (*n* = 200) received no osteoporosis education but were followed up with phone calls at 6 and 12 months. If they remained untreated at 12 months, they were offered the intensive intervention. Among the untreated patients at baseline, 18.8% in the no intervention group, 40.4% in the minimal intervention group and 53.2% in the intensive treatment group were treated at 12 months (*p* < 0.0001). Those who were already treated by their PCP at study baseline remained treated at 12 months, highlighting the importance of treatment initiation.

A cluster randomized controlled trial was conducted across small community hospitals in Ontario, involving patients > 40 years with a fragility fracture [80]. Patients attending hospitals allocated to the intervention arm (*n* = 130) were educated by a study coordinator who informed them of their risk of osteoporosis, importance of PCP follow-up and BMD testing, and provided an educational letter. A letter was also sent to their PCP highlighting the risk of osteoporosis and importance of BMD testing, recommendation of bisphosphonate treatment, pocket cards with Canadian guidelines, and provided specialist consultation if required. Patients randomized to the control arm (*n* = 137) received falls prevention education from the coordinator without mention of osteoporosis. The rate of osteoporosis treatment or prevention advice (if BMD normal) at 6 months was higher at 45% in the intervention group compared to 26% in the control group (*p* = 0.003). The rate of BMD testing was also higher in the intervention group (57% compared to 21%, respectively; *p* < 0.0001).

Another Canadian study evaluated the effect of a multifaceted intervention using a cluster randomized trial [81]. Postmenopausal women with a wrist fracture were recruited from emergency departments or hospital fracture clinics. Patients attending PCP practices randomized to the intervention group (*n* = 125) received a letter recommending a PCP visit to discuss osteoporosis and provided educational material and a checklist of fracture risk to bring to their appointment. Their PCP received a personalized letter informing them of their patient’s fracture, its association with osteoporosis and a two-page educational tool outlining Canadian guidelines. The patients of practices randomized to the control group (*n* = 145) received no communication. The intervention was shown to increase the proportion of women started on osteoporosis treatment (28% vs. 10%, *p* = 0.002) and referred for a BMD test (53.5% vs. 26%, *p* < 0.0001) compared to controls.

A large Quebec study covering numerous hospitals recruited women with fragility fractures and followed up after 6–8 months to assess diagnostic and treatment rates for osteoporosis [82]. Women were randomized to either (1) a ‘documentation group’ (*n* = 379) receiving written educational material on osteoporosis based on Canadian guidelines, stressing the importance of PCP follow-up to complete investigations and consider treatment; (2) a ‘video group’ (*n* = 409) receiving a 15-min educational video on osteoporosis with more in-depth discussion of osteoporosis-related topics in addition to written material; and (3) a control group (*n* = 386) receiving no further intervention. Investigation and treatment rates for osteoporosis remained low and only marginally higher in either intervention group (15–16% for BMD test and 11–12% for treatment) compared to the control group (12% for BMD test and 8% for treatment).

Another large Canadian study used medical claims data to identify patients over 50 years with a recent major fracture who had not previously undergone BMD testing or osteoporosis treatment [83]. Patients were randomized to either: (1) their PCP receiving a letter outlining osteoporosis management guidelines and an enclosed BMD test form (*n* = 1363), (2) the PCP receiving the same letter and BMD test form in addition to the patient receiving written information about osteoporosis (*n* = 1421) or (3) a control group receiving no intervention (*n* = 1480). At 1-year post-fracture, 16.4% in the PCP notification group, 18.2% in the PCP and patient notification group and 3.9% in the control group had BMD testing, while 14.7%, 16.5% and 10.6% had pharmacological treatment, respectively. This study showed significant improvements in the rate of osteoporosis investigations and treatment following a simple intervention.

Majumdar et al. recruited patients over 50 years with a wrist fracture at two large emergency departments in Alberta, using a non-randomized, ‘on–off’ study design where an intervention was delivered at one emergency department for 1 month while the other department received no intervention, switching over monthly [84]. The intervention (*n* = 55) consisted of a letter to the PCP reminding them of their patient’s wrist fracture, provided evidenced-based treatment guidelines including specific recommendation for bisphosphonates, and patient education through an information pamphlet and a telephone consultation within a week of the fracture. Control patients (*n* = 47) received falls prevention counselling and their PCP received standard discharge records of their fracture. At 6 months post-fracture, the intervention led to increased proportion of patients having a DXA scans compared to controls (62% vs. 17%, *p* < 0.001) and prescription of osteoporosis medications in those diagnosed with osteoporosis (40% vs. 10%, *p* = 0.002). At study conclusion, all controls were crossed over to intervention. Compared to the original intervention group, this delayed intervention resulted in equivalent scanning and treatment rates after 6 months [85]. The intervention strategy also led to cost-savings per patient and gain in quality-adjusted life years.

Several of the same researchers then demonstrated the efficacy of a case manager-led intervention in improving osteoporosis treatment rates in hip fracture patients over 50 years at 6 months post-fracture. This was not technically a PCP-directed study, as BMD testing and bisphosphonate prescription were initiated by the case manager rather than the PCP in the intervention group (*n* = 110) [86]. The control group (*n* = 110) also received educational material on osteoporosis, calcium and Vitamin D intake and falls prevention, which was more attentive than true ‘usual care’ in Canada. After 6 months, the control group became a ‘facilitated intervention group’ (*n* = 110), with the case manager only arranging for BMD testing, with results sent to the PCP to determine further management [87]. This less intensive intervention still improved rates of BMD testing (68% vs. 29%, *p* < 0.001) and bisphosphonate prescription (38% vs. 22%, *p* < 0.001) compared to usual care.

A study with a relatively short follow-up duration of 3 months showed that an intervention consisting of patient education and a letter to their PCP (*n* = 139) led to higher rates of BMD tests ordered but lower rates of BMD test performed and no difference in treatment initiation as compared to controls (*n* = 139) [88].

Three studies from the US showed that orthopaedic surgeons can also lead a primary care-focused intervention following a fragility fracture. In one small prospective study, 36 patients following a hip fracture were randomized to an intervention that included a 15-min orthopaedic-led inpatient discussion regarding the causal link between hip fractures and osteoporosis, the utility of DXA scans in its diagnosis, efficacy of bisphosphonate treatment and importance of PCP follow-up [89]. Patients were also provided with osteoporosis-related questions to take to their PCP with further reminders during a follow-up phone call 6 weeks post-discharge. The control group (*n* = 36) received written information on falls prevention containing a single mention of osteoporosis. At 6-month follow-up, 42% of patients had received a BMD test or commenced bisphosphonate therapy in the intervention group, compared to 19% in the control group (*p* = 0.036). In another study, fifty patients with a radius fracture were either randomized to the following: (1) the orthopaedic surgeon ordering, reviewing and sending the BMD test to the PCP (*n* = 27), or (2) sending a letter containing national guidelines recommending BMD testing to the PCP only (*n* = 23) [90]. At 6 months post-fracture, the group in the first intervention had a threefold increase in BMD testing (93% vs. 30%, *p* < 0.001) and osteoporosis treatment initiation (74% vs. 26%, *p* < 0.001). A similar study with 69 patients following a fragility fracture received verbal osteoporosis education and a letter to the PCP, resulting in a treatment rate of 56.4% and BMD testing rate of 43.6% out of those who followed up at 3 months [91].

The use of the electronic medical record in communicating with PCPs regarding secondary fracture prevention has been studied as early as 1999 in a US study involving patients over 50 years with a fragility fracture [92]. PCPs in the intervention group (*n* = 113) received EMR-based email communication informing them of the risk of osteoporosis and the need for further evaluation and potential treatment, along with access to management guidelines. By selecting the message, the PCP is taken to the patient’s record and can order further investigations, medications or contact the patient. A second patient-specific message was sent to PCPs who had not initiated further management after 3 months. Patients were also provided with educational osteoporosis material. This group was compared against a group where the PCP received above EMR notifications but no patient contact was made (*n* = 107) and a control group with no additional intervention (*n* = 107). At 6 months, 51.5% of the EMR plus patient group received BMD testing or osteoporosis treatment, compared to 43.1% in the EMR group and only 5.9% in the control group (*p* < 0.001). Integrating a patient-specific reminder into an EMR delivery system in the PCP’s workflow increased the likelihood of the PCP to recognize and act upon the advice.

#### Europe

In a UK study, patients admitted with a neck of femur fracture were targeted for a community intervention, whereby denosumab was written on the inpatient prescription chart, promoting inclusion in the final discharge summary. An additional consultant letter recommending denosumab prescription was separately sent to the PCP [93]. This led to an improvement of treatment initiation with denosumab from 22% in the pre-intervention phase to 74% post-intervention.

Naranjo et al. enrolled patients aged over 50 years with a fragility fracture who attended a Spanish emergency department in an observational study [94]. Patients (*n* = 330) were given an osteoporosis questionnaire, baseline DXA scan and education, and PCPs were trained in osteoporosis management. The primary outcome was adherence to oral bisphosphonate treatment in those who had been prescribed treatment at 3 months. A total of 67% of patients were recommended an oral bisphosphonate, of which 78% were still on treatment at 3 months follow-up.

The ESOSVAL is an osteoporosis study based in Valencia, Spain, aiming at improving osteoporosis care and reducing fracture risk through professional education of 800 participating PCPs and nurses, and optimizing the electronic record system with changes related to osteoporosis prevention and management [95]. One of the objectives of the ESOSVAL-F component of the study is to evaluate this multifaceted intervention on improvement rates of osteoporosis treatment between PCPs in the intervention arm (*n* = 400 primary care practices) compared to control practices (*n* = 400). Results from the decade-long follow-up are not yet available for review.

#### Middle East

A randomized controlled trial from Israel studied the effect of a PCP intervention in postmenopausal women following a distal radius fracture [96]. All women received a questionnaire and were informed about the possible link between the fracture and osteoporosis. The intervention group (*n* = 35) were given an explanatory pamphlet and a letter to their PCP while the control group (*n* = 35) received no further communication beyond the initial questionnaire. This intervention led to the proportion of patients undergoing osteoporosis workup to be 40% compared to 14.3% in controls (*p* < 0.001). Another intervention study from Israel utilized a nationwide osteoporosis registry and targeted treatment-naïve patients for both primary (T-score ≤  − 2.5 SD) or secondary prevention (previous hip or vertebral fracture) [97]. When the patient was reviewed by their physician (PCP, endocrinologist or geriatrician), they received alerts in the EMR from which referrals for blood tests, nutritionist, osteoporosis prescriptions and patient information print-outs could be directly ordered. Compared to the 3 years pre-intervention, time until treatment initiation in the post-intervention group decreased significantly, and initiation rates within 6 months increased from 12.3 to 27.7% and 17.4 to 27.1% among the hip and vertebral fracture cases, respectively (*p* < 0.001). PCPs who opened the ‘smart-set’ were more likely to initiate treatment than those who did not (40.5% vs. 24.1%, *p* < 0.001). Improvement in treatment initiation rates in the secondary prevention group were higher than the primary prevention group, and the authors postulated that ‘push alerts’ appear more useful in this setting, where fractures diagnosed in hospital settings are often missed during PCP review.

#### Asia–Pacific

Fracture patients were recruited from an Australian outpatient fracture clinic and contacted 3 months post-fracture [98]. At this point, over 80% of patients had no follow-up by their PCP. These patients were randomized to receive (1) a personalized letter outlining osteoporosis risk factors and recommending PCP follow-up (*n* = 75), or (2) the same letter in addition to an offer of a free BMD test (*n* = 79). In the group offered a BMD test, the proportion of patients investigated for osteoporosis was significantly higher than in the comparator group (38% vs. 7%, *p* = 0.001), leading to a diagnosis of osteopenia or osteoporosis in 67% of patients. However, treatment rates remained very low at 5–7% in both groups. This intervention highlighted that although the offer of a free BMD assessment led to improved rates of osteoporosis workup than a letter alone, this did not lead to higher treatment rates.

Currently, an Australian randomized controlled trial is underway which aims to evaluate whether alerting PCPs to their patient’s potential fragility fracture improves osteoporosis management. This novel model identifies potential fractures via natural language processing tools which screen radiology reports, rather than from hospital presentations. Patients over the age of 50 years with a potential osteoporotic fracture diagnosed through a private radiology practice or a hospital-based SFPP were included. PCPs working at practices randomized to the intervention (*n* = 600) will receive a fax and/or EMR alert informing them of their patient’s recent fracture along with management guidelines. The initial alert is then followed by a reminder survey to ascertain whether investigations or treatment has been initiated. PCPs working in practices randomized to the control arm (*n* = 600) will not receive such alerts. The difference in rates of BMD and blood testing, treatment initiation and continuation and chronic disease management plan initiation will be assessed through extensive data linkage.

### Summary of qualitative and quantitative studies

In summary, numerous studies have investigated strategies to improve the current osteoporosis care gap in primary care. Qualitative studies have consistently highlighted the importance of improving current methods of communication between hospitals and PCPs, recommending electronic delivery and integration with pre-existing software [54, 55, 75]. The role of the PCP in secondary fracture prevention remains disputed; some studies have highlighted the PCP preference for other specialists to initiate therapy [61, 76], whereas SFPPs find it difficult to transition patients to the PCP [77]. The interventions in quantitative studies largely focus on a combination of patient education, letters or EMR alerts to the PCP, telephone follow-up and ordering blood tests and/or bone mineral density tests. Overall, these studies demonstrate that targeted, intensive interventions with a two-pronged approach at educating both patients and PCPs lead to a significantly increased rate of osteoporosis treatment and/or BMD testing at study endpoints. Patient education was delivered either verbally over the phone, face-to-face in the hospital setting or via written educational material. One study examined whether including written patient education in additional to a PCP letter offered further advantage in rates of osteoporosis investigation and treatment, but the effect was marginal [83, 99]. Almost all the interventions involved a direct letter to the PCP either via physical copy or EMR, but no studies compared whether EMR provided an advantage over traditional methods of mail or fax. Studies which included EMR ‘push alerts’ and streamlined order sets showed significantly greater efficacy than other, less sophisticated approaches, pointing to a greater need for better technological integration in future studies [92, 97]. Interventions which arranged BMD testing independent of the PCP demonstrated significantly higher rates of BMD testing and osteoporosis treatment in two studies [87, 90] but no difference in treatment rate in one [98].

While these interventions demonstrate efficacy, they are clearly labour-intensive and require involvement from the hospital-based FLS team. The most intensively targeted group in the Roux et al. study required SFPP staff not only organizing verbal and written patient education and letter to the PCP, but also arranging blood and BMD tests, reviewing and flagging abnormal results to the PCP, following up patient phone calls at 3 time-points within 12 months and recontacting the PCP if no treatment has been initiated [79]. Such an intervention would be unfeasible at most SFPP centres. Finally, with potentially greater detection in the number of fractures through models utilizing natural language processing tools, the development of more automated and streamlined processes are required.

### Clinical standards for SFPPs and primary care

Clinical standards and key performance indicators, by which SFPPs are held accountable to, have been developed by numerous countries including Canada [100], the UK [101], Egypt [102], Japan, [103] and New Zealand [104]. There are also national registries that enable SFPPs to benchmark their performance, notably in Canada [105], USA [106], UK [107], Ireland [108], Australia [109] and New Zealand [110]. As an example, the Asia Pacific Consortium on Osteoporosis (APCO) Framework developed minimum clinical standards using a ‘5IQ’ model (identification, investigation, information, intervention, integration and quality) [111]. The purpose of such a framework is to provide clear recommendations for the care of osteoporosis in the region and guide healthcare policymakers in revising or developing guidelines.

While benchmarking of secondary (and primary) fracture prevention care typically occurs in the hospital setting, the ‘5IQ’ framework can also be applied to primary care settings and PCPs. Our narrative review has illustrated the potential for primary care to play a central role in secondary fracture prevention and identify a vast number of patients otherwise missed by hospital presentations alone. Therefore, applying similar, vigorous standards to the management of fracture prevention in primary care is essential, and future studies could examine how to incorporate the clinical standards into PCP-based software.

## Conclusion

With a rapidly ageing population, the global burden of osteoporosis will continue to grow. There is currently a significant hiatus in osteoporosis care, particularly affecting older patients at very high risk of fracture. In most countries, existing processes are inadequate to manage the increasing number of patients requiring secondary fracture prevention and management. While a proportion of these patients are managed in hospital-based SFPPs, these services are usually under-resourced and costly, and a shift towards primary care management is needed. Various studies have shown that osteoporosis is generally underdiagnosed and undertreated in primary care but that PCPs, when supported by hospital-based services, are clearly able and willing to effectively manage secondary fracture prevention, particularly with the aid of multifaceted interventions aimed at improving the hospital-primary care transition in osteoporosis care. However, currently more needs to be done to clearly define roles and responsibilities of healthcare providers, improve communication pathways between teams and provide additional education to patients and PCPs. There also needs to be better identification of patients needing secondary fracture prevention, not only from hospitals but also from community radiology practices. Greater involvement of other healthcare professionals such as community radiology practices, pharmacists, nurse practitioners and physiotherapists in models of care of secondary fracture prevention would also be beneficial. Further research, particularly randomized controlled trials which focus on interventions that integrate all of these processes, are urgently needed.
